# Tetragermanates of Strontium, Lead, and Barium of Formula Type AB_4_Q_9_

**DOI:** 10.6028/jres.065A.016

**Published:** 1961-04-01

**Authors:** Carl R. Robbins, Ernest M. Levin

## Abstract

Three new tetragermanates, SrGe_4_O_9_, PbGe_4_O_9_, and BaGe_4_O_9_, of formula type AB_4_O_9_ were found. BaTiGe_3_O_9_, the germanium analog of the mineral silicate benitoite (BaTiSi_3_O_9_) was prepared for study and comparison. Indexed X-ray powder diffraction patterns of the tetragermanates and of BaTiGe_3_O_9_ and BaTiSi_3_O_9_ show: (1) the tetragermanates are apparently isostructural; (2) the unit cell of BaTiGe_3_O_9_ at room temperature is related to that of the tetragermanates by a doubling of the *c*-axis of the latter; (3) the tetragermanates and the metastable (room temperature) form of BaTiGe_3_O_9_ are apparently structurally similar to, but not isostructural with benitoite; (4) within its temperature stability range, BaTiGe_3_O_9_ appears to be isostructural with BaTiSi_3_O_9_.

Density, melting point, and partial optical data for the tetragermanates were obtained.

## 1. Introduction

In the course of a study of the system SrO-GeO_2_ [1] 1 a new compound, SrGe_4_O_9_, of formula type AB_4_O_9_ was found.

Although the crystal chemistry of germanium is in many respects similar to that of silicon, no AB_4_O_9_ silicates are known. The mineral silicate benitoite, BaTiSi_3_O_9_, of formula type ABC_3_O_9_, one tetrasilicate [2] and three tetragermanates [3] of type A_2_B_4_O_9_, and a series of mixed oxides of formula type AB_2_C_2_O_9_ [4] have been reported. Barium tetratitanate, BaTi_4_O_9_ [5] was the only example of an AB_4_O_9_ compound found in the literature.

Accordingly, it was of interest to attempt to synthesize other AB_4_O_9_ germanates for study and comparison. In addition to SrO:4GeO_2_, mixtures in the ratios CaO:4GeO_2_, PbO:4GeO_2_, and BaO:4GeO_2_ were prepared for study. It was known from previous work [6] that the compound MgGe_4_O_9_ does not exist.

The germanium compositional analog of benitoite, BaTiGe_3_O_9_, had been synthesized previously [7], and natural crystals of BaTiSi_3_O_9_ were obtained for comparison with SrGe_4_O_9_ and any other AB_4_O_9_ germanates found.

## 2. Experimental Procedure

### 2.1. Materials and Methods

Homogeneous starting mixtures were prepared from reagent grade BaCO_3_, SrCO_3_, CaCO_3_, PbO, and GeO_2_ (quartz form). Mixtures were pressed into disks under a pressure of 10,000 psi. With the exception of the PbO :4GeO_2_ mixture, the disks were calcined for 2 to 3 hours in platinum crucibles at 1000 °C. The lead mixture was heated at temperatures up to 700 °C. The process of mixing, pressing and calcining was repeated 3 times.

The volatility of GeO_2_ and PbO is negligible at the calcining temperatures employed. Samples treated at higher temperatures were enclosed in sealed platinum capsules. Melting point determinations were made by the quenching method. Temperatures were measured with a calibrated Pt-Pt 10 percent Rh thermocouple. Samples were examined with binocular and polarizing microscopes and by X-ray powder diffractometry using Ni-filtered Cu radiation. High temperature X-ray studies were made with equipment described by Mauer and Bolz [8].

### 2.2. Density Determination

Since large crystals were not obtained in this study, the problem of determining the density of a small amount of fine powder was encountered. Approximate values were obtained with a Berman density balance on fragments of charges heated in sealed capsules for 4 hours. In addition, a theoretical optical density was calculated for each compound by applying the rule of Gladstone and Dale [9] to crystalline materials, as suggested by Larsen [10]. The specific refractivity formula of Gladstone and Dale is:

(n−1)/d=K,

where

*n* is the average index of refraction, *d* represents the density, and *K* is the sum of products of the weight fractions of the oxides by their respective specific refractivities.

The average index was measured or calculated from measured extreme indices using the relationship *n*=(*2ω*+*ϵ*)/3, the equation for uniaxial substances. Specific refractivity values were taken from Larsen [10] with the exception of the value of *k* = 0.165 for the *α*-quartz form of GeO_2_. The latter was calculated from the data of Laubengayer and Morton [11].

## 3. Results and Discussion

Three new compounds, SrGe_4_O_9_, PbGe_4_O_9_, and BaGe_4_O_9_, were found. Table 1 lists the density balance, optical and X-ray density values obtained. Melting point data, unit cell dimensions and optical properties are given in table 2.

CaGe_4_O_9_ was not formed at temperatures up to 1,000 °C, the maximum temperature to which the mixture was heated. X-ray diffraction patterns always showed GeO_2_ and an unidentified phase richer in CaO than the ratio CaO:4GeO_2_.

### 3.1. Description of Compounds

#### SrGe_4_O_9_

The compound SrGe_4_O_9_ melts congruently at 1298 ± 5 °C. It crystallizes in plates of irregular outline which show extremely low birefringence. Using the oil immersion method and white light, an average refractive index of 1.780 was observed. The optic sign could not be determined. The X-ray powder diffractometer pattern was indexed on the basis of a hexagonal unit cell with *a*=11.34A, *c*=4.75A, and *c*/*a*=0.4189. The theoretical X-ray density is 4.89 g/cm^3^ and there are 3 molecules per unit cell. X-ray data for the compound are given in table 3.

#### PbGe_4_O_9_

The compound PbGe_4_O_9_ was not observed by Speranskaya [12] in his recent study of the system PbO–GeO_2_. It dissociates at approximately 700 °C to GeO_2_ (rutile form) and an unidentified crystalline phase. Crystals of PbGe_4_O_9_ obtained were unsuitable for detailed optical study. They were birefringent with an average refractive index of approximately 1.800. The compound was indexed^2^ on a hexagonal cell with *a*=11.41A, *c*=4.75A, and *c*/*a*=0.4163. The X-ray density is 5.94 g/cm^3^ and Z=3. X-ray powder diffractometer data are given in table 4.

#### BaGe_4_O_9_

BaGe_4_O_9_ melts congruently at 1392 ±5 °C. Crystals obtained were platy with irregular, rounded shapes. Optical examination showed that the compound is uniaxial negative with *ω* =1.797 and *ϵ* = 1.783 (±.003). The X-ray powder diffraction pattern was indexed on the basis of a hexagonal unit cell with *a* = 11.61A, *c* = 4.74A and *c*/*a* = 0.4083. The theoretical X-ray density is 5.12 g/cm^3^ and there are 3 molecules per unit cell. X-ray powder diffraction data for the compound are given in table 5.

#### BaTiGe_3_O_9_

Goldschmidt [13] reported the synthesis of the compound BaTiGe_3_O_9_ at 1000 °C and claimed that it has the benitoite (BaTiSi_3_O_9_) structure type at room temperature.

The compound was studied by Bobbins [7] who found that BaTiGe_3_O_9_ is stable only from 1132 ±10 °C to 1255 ±10 °C. He concluded that the phase described by Goldschmidt as BaTiGe_3_O_9_ was apparently a solid solution of TiO_2_ in BaGe_4_O_9_. At room temperature, BaTiGe_3_O_9_ is apparently structurally similar to, but not isostructural with, BaTiSi_3_O_9_, having *a* = 11.73A, *c* = 10.02A and Z = 6. Within its temperature stability range, the compound appears to be isostructural with BaTiSi_3_O_9_, having *a* = 6.8A, *c* = 10.0A, and Z = 2. Room temperature X-ray diffraction data are given in table 6.

#### BaTiSi_3_O_9_

Zachariasen [14] determined the crystal structure of BaTiSi_3_O_9_. He found that the unit cell contained 2 molecules and had the following dimensions: *a*=6.60 ± 0.01A, *c* = 9.71 ± 0.01A. The space group is $D_{3h}^{2}\left(P\bar{6}\;c2\right)$ and the calculated density is 3.73 g/cm^3^.

**Figure 1:**
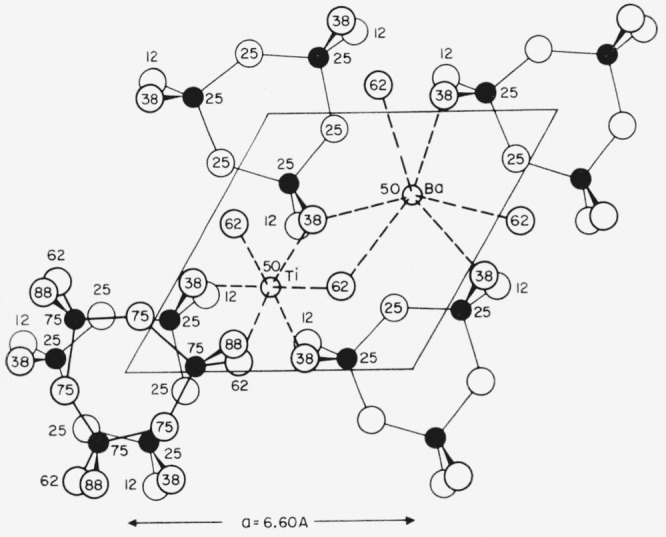
The structure of benitoite, *BaTiSi_3_O_9_* (after Bragg). Only atoms on either side of the reflection plane at height 25 are shown, except in the bottom lefthand corner. Superimposed oxygen atoms are symmetrically displaced. Note the Si_3_O_9_ rings.

The compound was interesting because it was the only known member of the ditrigonal-bipyramidal symmetry class, and because it afforded the only known example of SiO_4_ tetrahedra linked in rings of composition Si_3_O_9_. Subsequently, this structural group has been found in several other silicates. A projection of the structure (after Bragg [15]) on the 0001 plane is shown in figure 1. Both Ba and Ti are surrounded by 6 oxygen atoms. There are two layers of rings in the length of the *c*-axis.

X-ray powder diffractometer data (table 7) were obtained from natural crystals of BaTiSi_3_O_9_ from St. Benito Co., California (USNM #C3938). The compound was indexed on the basis of Zachariasen’s unit cell using the cell dimensions obtained in this study.

### 3.2. Structural Considerations

**Figure 2:**
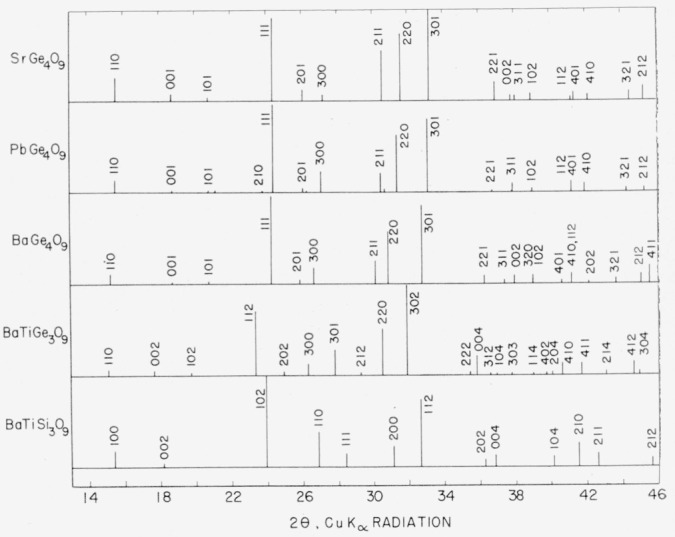
Schematic partial X-ray diffractometer patterns of the *AB_4_O_9_* Germanates, *BaTiGe_3_O_9_* (room temperature form) and *BaTiSi_3_O_9_*.

Unit cell dimensions of the AB_4_O_9_ germanates and of BaTiGe_3_O_9_ and BaTiSi_3_O_9_ are summarized in table 8, and schematic, partial X-ray diffraction patterns are shown in figure 2.

The AB_4_O_9_ germanates are apparently isostructural, having a *c*-axis of 4.75A and an *a*-axis varying from 11.34A for SrGe_4_O_9_ through 11.61A for BaGe_4_O_9_, with 3 molecules per unit cell. If certain reflections of the powder diffraction patterns are omitted, the 101, 201 and 211 for example, the AB_4_O_9_ germanates may be indexed on the benitoite (BaTiSi_3_O_9_) cell.

The AB_4_O_9_ germanates are related to the room temperature form (metastable) of BaTiGe_3_O_9_ by a doubling of the *c*-axis of the former. If certain reflections of the room temperature diffractometer pattern of BaTiGe_3_O_9_ are omitted, the 102, 202, and 212, for example, this form may be indexed on the benitoite cell. However, within its temperature stability range (1132° ± 10 °C to 1235° ± 10 °C), these reflections are not present, and BaTiGe_3_O_9_ is apparently isostructural with BaTiSi_3_O_9_. Corresponding reflections in the patterns of SrGe_4_O_9_ and BaGe_4_O_9_ did not disappear with heating, and no change was observed in the pattern of BaTiSi_3_O_9_ at temperatures up to 1160 °±20 °C.

Since the AB_4_O_9_ germanates appear to be structurally very similar to BaTiSi_3_O_9_, rings of GeO_4_ tetrahedra of composition Ge_3_O_9_ are most probably their dominant structural unit. Since germanium may have either 4-fold or 6-fold coordination with respect to oxygen, the fourth germanium atom would occupy the titanium position of BaTiSi_3_O_9_, in octahedral coordination. Thus the formula BaGe_4_O_9_ should, perhaps more correctly, be written as BaGeGe_3_O_9_.

The observed differences between BaTiGe_3_O_9_, BaTiSi_3_O_9_ and the AB_4_O_9_ germanates result primarily from a difference in size between Ti and Ge (in octahedral coordination) and between Si and tetrahedrally coordinated Ge. In the lower left corner of figure 1 it is seen that the upper Si_3_O_9_ ring does not lie directly above the lower one, and two units are required in the length of the *c*-axis of BaTiSi_3_O_9_. A similar rotation of a corresponding Ge_3_O_9_ ring presumably is the reason for the doubling of the *c*-axis of the BaGe_4_O_9_ structure when one Ti is substituted for one Ge to form BaTiGe_3_O_9_. It should be noted that the long diagonal of figure 1 is of the order of magnitude of the 11A *a*-axis of the AB_4_O_9_ germanates and metastable BaTiGe_3_O_9_. Apparently the substitution of the larger Ge_3_O_9_ groups for Si_3_O_9_ groups requires that the diagonal of the silicate cell become the *a*-axis of the germanate cell. Within the temperature stability range of BaTiGe_3_O_9_ this distortion is relieved, and the compound has the 6A *a*-axis of the silicate.

Preliminary intensity calculations indicate that the preceding structural assumptions for the AB_4_O_9_ germanates are correct. Suitable single crystals of BaGe_4_O_9_ have been obtained, and a detailed study of the structure of the compound is in progress.

## 4. Summary

Three new compounds, SrGe_4_O_9_, PbGe_4_O_9_ and BaGe_4_O_9_, of formula type AB_4_O_9_, have been synthesized. These tetragermanates appear to be isostructural. They are apparently structurally similar to, but not isostructural with, BaTiSi_3_O_9_.

The compound BaTiGe_3_O_9_ is metastable at room temperature. The metastable form is related to the AB_4_O_9_ germanates by a doubling of the *c*-axis of the latter. Metastable BaTiGe_3_O_9_ is apparently structurally similar to, but not isostructural with, BaTiSi_3_O_9_. It appears to be a structural link between the AB_4_O_9_ germanates and BaTiSi_3_O_9_, having the 11A *a*-axis of the former, and the two layers of rings in the length of the *c*-axis of the latter.

Within its temperature stability range, BaTiGe_3_O_9_ is apparently isostructural with BaTiSi_3_O_9_.

## Figures and Tables

**Table 1:** A comparison of density values for the *AB_4_O_9_* germanates

Compound	Density Balance	Optical	X-Ray
			
	(*g/cm*^3^)	(*g/cm*^3^)	(*g/cm*^3^)
SrGe_4_O_9_	a4.84	4.85	4.89
PbGe_4_O_9_	b5.89	5.96	5.94
BaGe_4_O_9_	a5.07	5.11	5.12

a Sintered at 1200 °C for 4 hours.

b Sintered at 650 °C for 4 hours.

**Table 2:** Some properties of the *AB_4_O_9_* germanates

Compound	Melting Point	Unit Cell Dimensions	*Z*	X-Ray Density	Optical Properties		
					Character	Indices	*n* of glass
							
	*°C*	*A*		(*g/cm*^3^)		(±.003)	(±.003)
SrGe_4_O_9_	1298±5°	Hex. a=11.34 Hex. c=4.75	3	4.89	weakly birefringent	$\bar{\eta }=1.780$	1.735
PbGe_4_O_9_	dissociates at ~ 700°.	Hex. a=11.41 Hex. c=4.75	3	5.94	anhedral, birefringent	$\bar{\eta }=1.800$	1.776
BaGe_4_O_9_	1392±5°	Hex. a=11.61 Hex. c=4.74	3	5.12	anhedral, birefringent, uniaxial negative.	*ω* = 1.797 *ϵ* = 1.783	1.746

**Table 3:** X-ray^a^ powder diffraction data for *SrGe_4_O_9_*

*hkl*	*d*obs	*I/I*_0_^b^	$\frac{1}{d^{2}\text{obs}}$	$\frac{1}{d^{2}\text{cal}}$^c^
				
	*A*			
110	5.67	26	0.0311	0.0311
001	4.75	8	.0443	.0443
101	4.28	5	.0547	.0546
111	3.65	92	.0752	.0754
201	3.42	13	.0857	.0858
300	3.27	7	.0933	.0934
211	2.925	55	.1169	.1169
220	2.834	74	.1245	.1245
301	2.695	100	.1377	.1376
221	2.435	21	.1686	.1688
002	2.377	6	.1770	.1770
311	2.364	6	.1789	.1791
102	2.310	8	.1874	.1874
112	2.191	4	.2083	.2082
401	2.182	9	.2101	.2103
410	2.143	7	.2178	.2179
321	2.036	10	.2414	.2414
212	2.002	15	.2495	.2497
411	1.9544	13	.2618	.2621
302	1.9239	6	.2702	.2704
330	1.8960	2	.2799	.2801
222	1.8219	19	.3013	.3015
312	1.7918	8	.3115	.3119
331	1.7568	14	.3240	.3244
402	1.7077	4	.3429	.3430
600	1.6372	14	.3731	.3735
412	1.5923	4	.3944	.3949

a Cu K*α* Radiation.

b *I/I*_0_ represents the intensity of the diffraction peak relative to the strongest peak.

c Calculated on the basis of the following unit cell dimensions: *a*=11.34A, *c*=4.75A.

**Table 4:** X-ray^a^ powder diffmction data for *PbGe_4_O_9_*

*hkl*	*d*obs	$I_{/I_{0}}$^b^	$\frac{1}{d^{2}\text{obs}}$	$\frac{1}{d^{2}\text{cal}}$^c^
				
	*A*			
110	5.70	14	0.0308	0.0307
001	4.74	2	.0445	.0444
101	4.28	1	.0547	.0546
…	d4.20	1	…	…
210	3.74	2	.0717	.0717
111	3.65	100	.0751	.0751
201	3.42	5	.0855	.0854
…	d3.39	2	…	…
300	3.29	23	.0923	.0922
211	2.932	22	.1163	.1161
…	d2.914	4	…	…
220	2.851	62	.1230	.1230
301	2.704	79	.1368	.1366
221	2.444	1	.1674	.1674
311	2.373	10	.1777	.1776
102	2.307	4	.1879	.1878
112 401	} 2.190	12	.2085	.2083
410	2.155	10	.2153	.2152
321	2.044	4	.2393	.2391
212	2.002	5	.2495	.2493
411	1.9628	25	.2596	.2596
302	1.9255	10	.2697	.2697
330	1.9008	4	.2768	.2767
501 222	} 1.8239	21	.3006	$\left\{ \begin{array}{l} .3006\\ .3005 \end{array}\right.$
312	1.7934	5	.3109	.3107
331	1.7647	23	.3211	.3211
421	1.7372	1	.3314	.3313
402	1.7113	3	.3415	.3415
511	1.6609	1	.3623	.3621
600	1.6464	14	.3689	.3689

a CuK*α* radiation.

b $I_{/I_{0}}$ represents the intensity of the diffraction peak relative to the strongest peak.

c Calculated on the basis of the following unit coll dimensions: *a*=11.41A, *c*=4.75A.

d See discussion of PbGe_4_O in text.

**Table 5:** X-ray^a^ powder diffraction data for *BaGe_4_O_9_*

*hkl*	*d*obs	$I_{/I_{0}}$^b^	$\frac{1}{d^{2}\text{obs}}$	$\frac{1}{d^{2}\text{cal}}$^c^
	*A*			
110	5.80	11	0.0298	0.0297
001	4.74	2	.0446	.0446
101	4.28	3	.0546	.0545
111	3.67	100	.0743	.0743
201	3.44	6	.0843	.0842
300	3.35	17	.0893	.0891
211	2.963	25	.1139	.1139
220	2.899	56	.1190	.1188
301	2.733	86	.1339	.1337
221	2.473	9	.1635	.1634
311	2.401	4	.1735	.1733
002	2.368	8	.1783	.1783
320 102	} 2.306	9	.1881	$\left\{ \begin{array}{l} .1861\\ .1882 \end{array}\right.$
401	2.219	4	.2032	.2029
410 112	}2.192	11	.2081	.2079
202	2.142	2	.2180	.2179
321	2.072	5	.2329	.2326
212	2.010	10	.2475	.2475
411	1.9893	18	.2527	.2524
330 302	}1.9344	11	.2673	.2673
501	1.8499	2	.2922	.2920
222	1.8349	22	.2970	.2970
312 510	} 1.8046	6	.3070	.3069
331	1.7905	25	.3119	.3118
402	1.7232	2	.3368	.3366
600	1.6746	17	.3566	.3563

a Cu K*α* radiation.

b $I_{/I_{0}}$ represents the intensity of the diffraction peak relative to the strongest peak.

c Calculated on the basis of the following unit cell dimensions: *a* = 11.61 A, *c*=4.74A.

**Table 6:** X-ray^a^ powder diffraction data for *BaTiGe_3_O_9_* b at room temperature

*hkl*	*d*obs	$I_{/I_{0}}$^c^	$\frac{1}{d^{2}\text{obs}}$	$\frac{1}{d^{2}\text{cal}}$^d^
				
	*A*			
110	5.85	7	0.0292	0.0291
002	5.01	6	.0398	.0398
102	4.49	3	.0496	.0495
112	3.81	65	.0690	.0689
202	3.57	5	.0787	.0786
300	3.39	13	.0872	.0872
301	3.21	28	.0973	.0972
212	3.05	3	.1078	.1077
220	2.928	50	.1166	.1163
302	2.803	100	.1273	.1271
222	2.531	4	.1561	.1561
004	2.505	21	.1594	.1594
312	2.453	2	.1662	.1658
104	2.430	2	.1694	.1691
303	2.378	2	.1769	.1769
114	2.305	16	.1882	.1884
402	2.264	3	.1951	.1949
204	2.247	3	.1981	.1981
410	2.218	13	.2033	.2035
411	2.164	13	.2135	.2135
214	2.097	4	.2274	.2272
412	2.028	14	.2432	.2433
304	2.015	3	.2463	.2466
330	1.956	7	.2615	.2616
224	1.905	15	.2756	.2757
312	1.874	3	.2849	.2853
413	1.848	6	.2928	.2931
332	1.822	11	.3012	.3015

a Cu K*α* Radiation.

b This specimen was prepared by heating a portion of the calcined starting mixture in a sealed platinum capsule at 1150 °C for 5 days, and quenching it.

c *I*/*I*_0_ represents the intensity of the diffraction peak relative to the strongest peak.

d These values were calculated on the basis of the following unit cell dimensions: *a* = 11.73A and *c* = 10.02A.

**Table 7:** X-ray a powder diffraction data for benitoite (*BaTiSi_3_O_9_*) b

hkl	*d*obs	I/I_0_^c^	$\frac{1}{d^{2}\text{obs}}$	$\frac{1}{d^{2}\text{cal}}$^d^
	*A*			
100	5.74	18	0.0304	0.0302
002	4.87	5	.0421	.0421
102	3.72	100	.0724	.0723
110	3.32	38	.0909	.0907
111	3.14	14	.1014	.1012
200	2.873	23	.1211	.1210
112	2.742	74	.1330	.1328
202	2.475	8	.1633	.1630
004	2.438	12	.1683	.1683
104	2.245	12	.1985	.1986
210	2.172	25	.2120	.2117
211	2.120	14	.2225	.2222
212	1.984	9	.2540	.2538
114	1.965	28	.2590	.2590
300	1.916	21	.2723	.2722
204	1.859	20	.2894	.2893
302	1.783	17	.3147	.3143
220	1.659	4	.3632	.3629
214	1.622	12	.3801	.3800
310	1.594	4	.3937	.3931
222	1.571	4	.4053	.4050
106	1.567	11	.4072	.4072

a Cu K*α* radiation.

b Data obtained from natural crystals of BaTiSi_3_O_9_ from St. Benito Co., California (USNM #C3938).

c I/I_0_ represents the intensity of the diffraction peak relative to the strongest peak.

d Calculated on the basis of following unit cell dimensions: *a*=6.64A, *c*=9.75A. Zachariasen [14] reported the following values: *a*=6.60±0.01A, *c*=9.71±0.01A.

**Table 8:** A comparison of the unit cell dimensions of the *AB_4_O_9_* germanates and of *BaTiGe_3_O_9_* and *BaTiSi_3_O_9_*

Compound	*a*	*c*	Z
			
	*A*	*A*	
SrGe_4_O_9_	11.34	4.75	3
PbGe_4_O_9_	11.41	4.75	3
BaGe_4_O_9_	11.61	4.74	3
BaTiGe_3_O_9_^a^	11.73	10.02	6
BaTiGe_3_O_9_^b^	6.8	10.0	2
BaTiSi_3_O_9_^c^	6.60	9.71	2

a Measured at room temperature.

b Measured at 1160 °±20 °C.

c Zachariasen’s values [14].

## Footnotes

1 Figures in brackets indicate the literature references at the end of this paper.

2 Three very weak diffraction peaks, not indexable on the basis of this cell, were observed. Preliminary studies of the system PbO–GeO_2_ suggest that they represent a trace of metastably formed PbGeCO_3_.

## References

[b1-jresv65an2p127_a1b] 1C. R. Robbins and E. M. Levin, The System SrO-GeO_2_, paper in preparation.

[2] Kracek FC, Bowen NL, Morey GW (1937). Equilibrium relations and factors influencing their determination in the system K_2_SiO_3_-SiO_2_. J Phys Chem.

[3] (1931). Gmelins Handbuch.

[4] Aurivillius Bengt (1950). Mixed bismuth oxides with layer lattices. ARKIV Für Kemi.

[5] Rase DE, Roy R (1955). Phase equilibrium in the system BaO-TiO_2_. J Am Ceramic Soc.

[6] Robbins CR, Levin EM (1959). The system magnesium oxide-germanium dioxide. Am J Sci.

[7] Robbins CR The compound BaTiGe_3_O_9_. J Am Ceramic Soc.

[8] Mauer FA, Bolz LH (1953). Thermal expansion of Cermet components by high temperature X-ray diffraction.

[9] Gladstone JH, Dale TP (1864). Researches on the refraction, dispersion, and sensitiveness of liquids. Roy Soc London Philos Trans.

[10] Larsen ES, Berman H (1934). The microscopic determination of the nonopaque minerals.

[11] Laubengayer AW, Morton DS (1932). The polymorphism of germanium dioxide. J Am Chem Soc.

[12] Speranskaya EI (1959). System PbO-GeO_2_.

[13] Goldschmidt VM (1931). Zur Kristallchemie des Germaniums.

[14] Zachariasen WH (1930). The crystal structure of benitoite, BaTiSi_3_O_9_. Z Krist.

[15] Bragg WL (1937). Atomic Structure of Minerals.

